# PGE_2_ maintains self-renewal of human adult stem cells via EP2-mediated autocrine signaling and its production is regulated by cell-to-cell contact

**DOI:** 10.1038/srep26298

**Published:** 2016-05-27

**Authors:** Byung-Chul Lee, Hyung-Sik Kim, Tae-Hoon Shin, Insung Kang, Jin Young Lee, Jae-Jun Kim, Hyun Kyoung Kang, Yoojin Seo, Seunghee Lee, Kyung-Rok Yu, Soon Won Choi, Kyung-Sun Kang

**Affiliations:** 1 Adult Stem Cell Research Center, College of Veterinary Medicine, Seoul National University, Seoul 08826, South Korea; 2 Pusan National University School of Medicine, Busan 49241, South Korea; 3 Biomedical Research Institute, Pusan National University Hospital, Busan 49241, South Korea; 4 Research Institute for Veterinary Medicine, College of Veterinary Medicine, Seoul National University, Seoul 08826, South Korea; 5 Hematology Branch, National Heart, Lung and Blood Institute, National Institutes of Health, Bethesda, MD 20892, USA

## Abstract

Mesenchymal stem cells (MSCs) possess unique immunomodulatory abilities. Many studies have elucidated the clinical efficacy and underlying mechanisms of MSCs in immune disorders. Although immunoregulatory factors, such as Prostaglandin E_2_ (PGE_2_), and their mechanisms of action on immune cells have been revealed, their effects on MSCs and regulation of their production by the culture environment are less clear. Therefore, we investigated the autocrine effect of PGE_2_ on human adult stem cells from cord blood or adipose tissue, and the regulation of its production by cell-to-cell contact, followed by the determination of its immunomodulatory properties. MSCs were treated with specific inhibitors to suppress PGE_2_ secretion, and proliferation was assessed. PGE_2_ exerted an autocrine regulatory function in MSCs by triggering E-Prostanoid (EP) 2 receptor. Inhibiting PGE_2_ production led to growth arrest, whereas addition of MSC-derived PGE_2_ restored proliferation. The level of PGE_2_ production from an equivalent number of MSCs was down-regulated via gap junctional intercellular communication. This cell contact-mediated decrease in PGE_2_ secretion down-regulated the suppressive effect of MSCs on immune cells. In conclusion, PGE_2_ produced by MSCs contributes to maintenance of self-renewal capacity through EP2 in an autocrine manner, and PGE_2_ secretion is down-regulated by cell-to-cell contact, attenuating its immunomodulatory potency.

MSCs are potential candidates for the treatment of immune disorders such as graft-versus-host disease, rheumatoid arthritis, inflammatory bowel disease and multiple sclerosis^1^. Recently, many researchers have elucidated the safety and distinct functions related to the therapeutic application of MSCs, including paracrine factor-mediated immunomodulatory ability and stemness, which is defined as exhibiting stem cell properties represented by the ability to generate daughter cells identical to themselves (self-renewal) and to differentiate into multiple cell lineages (multipotency)^2^. Although a number of researchers have established methods for expanding MSCs in the laboratory and uncovered most of the mechanisms underlying MSC stemness, further studies are required to develop the most efficient procedure to harvest sufficient numbers of stem cells and to fully elucidate any unknown mechanisms for therapeutic application^3^. Moreover, the development of novel approaches to improve the therapeutic efficacy of MSCs is a major topic in the MSC research field. To improve therapeutic efficacy, several groups have manipulated the cells by pre-treating MSCs with growth factors and cytokines or by genetic modification^4^,^5^. However, these approaches are controversial because the precise mechanisms based on selected candidate factors such as NO, IDO, IL-10, and PGE_2_ from MSCs in specific diseases are not yet fully described. To address these issues, more detailed studies are required to explore the production and functions of candidate factors individually and link their function with the cellular properties.

PGE2 is a subtype of the prostaglandin family, which includes lipid mediators with physiological effects such as uterine contraction, cervix softening, fever induction, muscle relaxation and vasodilation. PGE_2_ is synthesized from arachidonic acid (AA) released from membrane phospholipids through sequential enzymatic reactions. Cyclooxygenase-2 (COX-2), known as prostaglandin-endoperoxidase synthase, converts AA to prostaglandin H_2_ (PGH_2_), and PGE_2_ synthase isomerizes PGH_2_ to PGE_2_^6^. As a rate-limiting enzyme, COX-2 controls PGE_2_ synthesis in response to physiological conditions, including stimulation by growth factors, inflammatory cytokines and tumour promoters^7^,^8^. PGE_2_ is secreted to the extracellular environment by multidrug-resistant protein 4 (MRP4)-mediated active transport and binds to specific EP receptors on target cells^9^. EP receptor is a G-protein coupled receptor (GPCR), and these receptors can be classified into 4 subclasses. EP2 receptor enhances cell proliferation and neovascularisation by increasing vascular endothelial growth factor (VEGF) secretion in several cancers^7^,^10^,^11^. In contrast, EP3 receptor-mediated signalling regulates cell proliferation by decreasing cAMP levels, consequently suppressing tumour development. In tumour-progressing cells, EP2 receptor is highly expressed, while the EP3 receptor expression level is relatively low^12^,^13^. This COX-2/PGE_2_ axis forms an autocrine/paracrine loop, affecting the cell cycle and apoptosis to regulate cell proliferation and viability via the activation of one or more EP receptors^14^. Using several *in vitro* and *in vivo* models of immune disorders, including Crohn’s disease and atopic dermatitis, we have shown that COX-2 signalling and PGE_2_ production in MSCs are crucial factors in the immunomodulatory ability of hMSCs^15^,^16^,^17^,^18^,^19^. Therefore, studies investigating the detailed regulatory mechanisms that focus on PGE_2_ production and function in MSCs are required to further develop therapeutic approaches.

Most eukaryotic cells assemble and construct 3D structures in organs, communicating with each other in response to intra- and extracellular stimuli. Gap junctions form intercellular connections via membrane-incorporated hexamers composed of connexin proteins in cell-to-cell contact. They control cell death and electrophysiology by delivering electrical currents, ions and small molecules. Connexin 43 (CX43) protein expression and gap junction intercellular communication (GJIC) were augmented by PGE_2_ produced by mechanical stress via EP2 receptor signalling in an autocrine manner^20^. However, the GJIC-mediated regulation of the COX-2/PGE_2_ axis is not yet reported.

In the present study, we assessed the role of PGE_2_ produced by human adult stem cells in the regulation of self-renewal and immunomodulation in an autocrine/paracrine manner using MSCs from two different sources, umbilical cord blood and adipose tissue. Furthermore, this study was designed to reveal the regulatory mechanism of PGE_2_ production in adult stem cells by gap junction intercellular communication (GJIC) when intimate cell-to-cell contact is allowed. Given that the basal level of PGE_2_ synthesis in human bone marrow-derived MSCs (hBM-MSCs) is significantly lower than in human umbilical cord blood-derived MSCs (hUCB-MSCs) or human adipose tissue-derived MSCs (hAD-MSCs), as proven in our previous study, we used hUCB-MSCs and hAD-MSCs in the present study to generalize PGE_2_-mediated regulation of adult stem cell functions. Moreover, hBM-MSCs are larger than hUCB-MSCs or hAD-MSCs, making it difficult to include hBM-MSCs in the determination of cell proliferation and secretion under the same experimental environment. Therefore, we generalized the PGE_2_-mediated novel properties of human adult stem cells using hUCB-MSCs and hAD-MSCs.

## Results

### Proliferation of human adult stem cells is decreased by the inhibition of COX-2 or mPGES-1 via G_1_ cell cycle arrest

**Figure 1:**
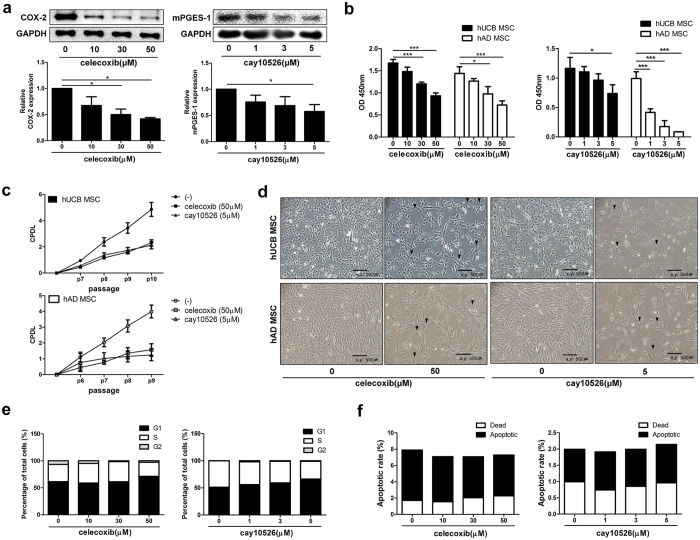
Inhibition of COX-2/mPGES-1 reduces the cellular growth of hUCB-MSCs and hAD-MSCs through G_1_ cell cycle arrest. hUCB-MSCs and hAD-MSCs were treated with celecoxib (selective inhibitor for COX-2) or cay10526 (selective inhibitor for mPGES-1) at indicated concentrations. (**a**) COX-2 and mPGES-1 protein levels in hUCB-MSCs were examined by Western blot analysis. Cell proliferation was determined by (**b**) bromodeoxyuridine (BrdU) assay and (**c**) CPDL. (**d**) Phase-contrast images, bar = 500 μm. Upper panel: hUCB-MSCs. lower panel: hAD-MSCs. ▼; flattened or spread out cell bodies. (**e**) FACS analysis of cell cycle and (**f**) apoptosis. Gel electrophoresis was conducted under the same experimental conditions, and images of blots were cropped. Uncropped blot images are shown in Suppl. Fig. S5. Results show a representative experiment. *P < 0.05, ***P < 0.001. Results are shown as the mean ± SEM.

**Figure 2:**
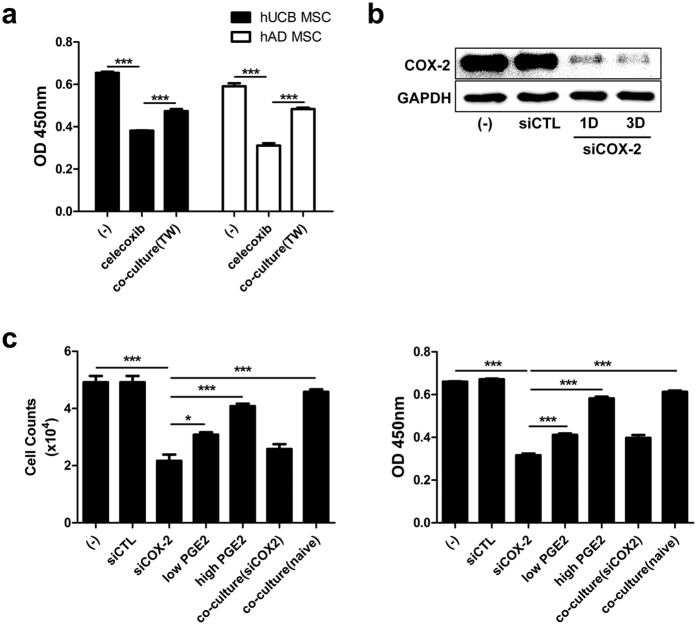
PGE_2_ produced by hMSCs restores the COX-2-mediated inhibition of cell proliferation. (**a**) COX-2 inhibited cells were co-cultured with naive cells for 24 hours, and the proliferation was determined by BrdU assay. After siRNA transfection, (**b**) sustained expression levels of COX-2 on day 1 and 3 were detected by Western blot analysis. (**c**) siCOX-2 transfected cells were treated with PGE_2_ or co-cultured with naive and COX-2 suppressed cells, and proliferation was measured by direct cell counts and BrdU assay. Gel electrophoresis was conducted under the same experimental conditions, and images of blots were cropped. *P < 0.05, ***P < 0.001. Results are shown as the mean ± SEM.

Indomethacin, the inhibitor for both COX-1 and COX-2, interferes with epithelial and tumour cell growth^21^,^22^. Therefore, we first investigated whether COX inhibition affected the proliferation of hUCB-MSCs and found that indomethacin treatment significantly decreased hUCB-MSC proliferation (Suppl. Fig. S1). In previous studies, similar effects were observed when COX-2 was selectively inhibited in other cell types^23^,^24^. We next investigated whether selective inhibition of COX-2 using celecoxib or inhibition of membrane associated PGE synthase 1 (mPGES-1), a PGE_2_ synthesizing enzyme downstream of COX-2 signalling, using cay10526 affected the proliferative phenotype of hUCB-MSCs and hAD-MSCs. Treatment down-regulated the expression of COX-2 or mPGES-1 at the protein level in a dose-dependent manner (Fig. 1a) Consistent with the decrease in the protein level, inhibition of PGE_2_ producing enzymes resulted in the remarkable dose-dependent decrease in the proliferation of both hUCB-MSCs and hAD-MSCs (Fig. 2b). The cumulative proliferative phenotype in MSCs in response to chemical inhibitors was further confirmed by evaluating the cumulative population doubling level (Fig. 1c). When COX-2 expression was inhibited by small interfering RNA (siRNA), the same results were observed (Suppl. Fig. S1). In addition, we showed that this suppression of self-renewal influenced cell confluency and cellular morphology. Compared with non-treated cells, celecoxib or cay10526-treated cells exhibited flattened or spread out cell bodies with low confluency (Fig. 1d, marked as ▼). To determine whether these changes in proliferation resulted from the lack of PGE_2_, the PGE_2_ secretion level was measured by enzyme-linked immunosorbent assay (ELISA). Conditioned media (CM) from COX-2-suppressed hUCB-MSCs contained less than 20% of the PGE_2_ concentration of the naïve MSC group (Suppl. Fig. S1). Inhibition of PGE_2_ production led to lower proliferation via G_1_ cell cycle arrest. The proportion of cells in G_1_ phase gradually increased dose-dependently, whereas the proportion of cells in S phase decreased (Fig. 1e). The apoptotic rate of hUCB-MSCs was not affected by the suppression of PGE_2_ synthesis (Fig. 1f and Suppl. Fig. S1). We next investigated whether the suppression of COX-2/PGE_2_ axis influence the other properties of MSCs. The expression pattern of surface antigens on hUCB-MSC was not altered after the treatment of celecoxib or cay10526 (Suppl. Fig. S2). Moreover, the expression levels of pluripotency marker genes in hUCB-MSCs were not significantly changed by the inhibition of the COX-2 (Suppl. Fig. S2). In addition, we found that up-regulation of COX-2/PGE_2_ signalling enhanced osteogenesis of hUCB-MSCs, in contrast, suppressed adipogenesis using RT-PCR and specific staining after the induction of differentiation. There was no significant change in chondrogenic differentiation (Suppl. Fig. S2). Taken together, these findings indicate that the COX-2/PGE_2_ axis has a critical role in the maintenance of hMSC self-renewal and that down-regulation of this axis leads to cell cycle arrest in G1 phase without affecting cell apoptosis.

### Decreased cell proliferation by COX-2 inhibition is restored by soluble factors from naive hMSCs

We next examined whether PGE_2_ produced by naive hMSCs could restore the proliferation of COX-2-suppressed hMSCs. To determine the effect of soluble factors from naïve hMSCs on COX-2-inhibited cells without cell-to-cell contact, hMSCs were treated with celecoxib for 3 days and subsequently co-cultured with naive cells for 24 hours using the transwell system. Interestingly, co-culture with intact cells rescued the cell growth rate of both celecoxib-treated hUCB-MSCs and hAD-MSCs (Fig. 2a). However, celecoxib-mediated COX-2 inhibition was restored as early as day 3 after treatment (Suppl. Fig. S3). To minimize the influence of the short duration of COX-2 inhibition, COX-2 was further inhibited by siRNA transfection. Specific siRNA for COX-2 (siCOX-2) stably inhibited the expression level of COX-2 until day 3 (Fig. 2b). Therefore, we further investigated whether the soluble factor, assumed to be PGE_2_, from naïve hMSCs restored the proliferation of COX-2-supressed hMSCs using siCOX-2 to induce relatively persistent inhibition. As expected, soluble factors from intact hUCB-MSCs significantly rescued the proliferation of COX-2-inhibited hUCB-MSCs, whereas secretory factors from siCOX-2-treated hUCB-MSCs did not (Fig. 2c). Moreover, direct PGE_2_ treatment increased the proliferation of COX-2-inhibited hUCB-MSCs in a dose-dependent manner (Fig. 2c). These results suggest that PGE_2_ exerts an autocrine regulatory function in the self-renewal of hMSCs.

### EP2 receptor is involved in autocrine PGE_2_ signalling to regulate hMSC proliferation

**Figure 3:**
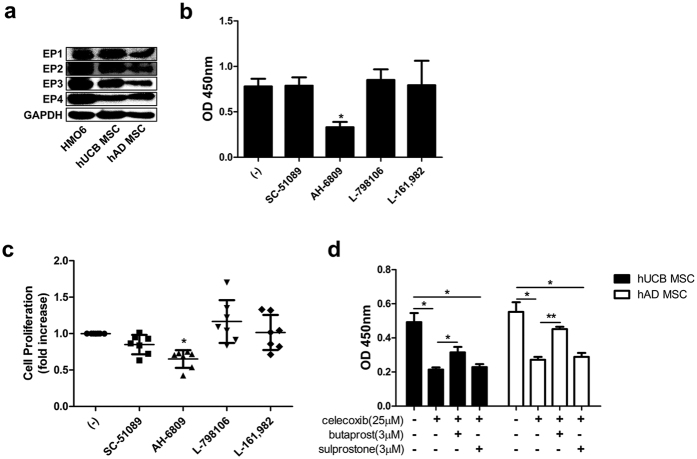
EP2 receptor has a crucial role in hMSC self-renewal. (**a**) EP receptor expression in hUCB-MSCs and hAD-MSCs was assessed by Western blot analysis. HMO6, a human microglia cell line, was used as a positive control. In the presence of selective blockers for EPs, (**b**) cell growth rates were examined by BrdU assay and (**c**) results of repeated experiments are presented. Selective blockers for EP1: SC-51089, EP2: AH-6809, EP3: L-798106, and EP4: L-161,982. (**d**) After celecoxib treatment, cells were treated with selective agonists for EP2 or EP3, and proliferation was measured with a BrdU assay kit. Gel electrophoresis was conducted under the same experimental conditions, and images of blots were cropped. Selective agonist for EP2: Butaprost, EP3: Sulprostone. *P < 0.05, **P < 0.01. Results are shown as the mean ± SEM.

PGE_2_ has a regulatory role in the self-renewal of hMSCs, and receptor-mediated signalling is involved in this regulation, including through EP receptors^25^,^26^. Therefore, we examined which receptors are expressed in hMSCs. Because microglia express all EP receptor subtypes, the human microglia cell line HMO6 was used as a positive control. hUCB-MSCs and hAD-MSCs expressed four EP receptor subtypes (Fig. 3a). We next explored the crucial receptors involved in PGE_2_-mediated cell growth regulation by blocking each receptor with selective antagonists. Remarkably, hUCB-MSC proliferation decreased only when the EP2 receptor was blocked with its antagonist (AH-6809) (Fig. 3b,c). The other antagonists did not affect proliferation significantly and EP3 antagonist, L-798106, slightly increased the proliferation (Fig. 3b,c). To confirm these findings, hMSCs were treated with butaprost and sulprostone (agonists for EP2 and EP3 receptors) in the presence of celecoxib to determine the effect of specific receptor triggering. While EP2 receptor activation significantly enhanced hUCB-MSC proliferation, EP3 receptor activation did not (Fig. 3d). Taken together, these results indicate that the EP2 prostanoid receptor is the pivotal signalling pathway in PGE_2_-mediated regulation of hMSC self-renewal.

### PGE_2_ secretion by hMSCs is regulated by cell contact

**Figure 4:**
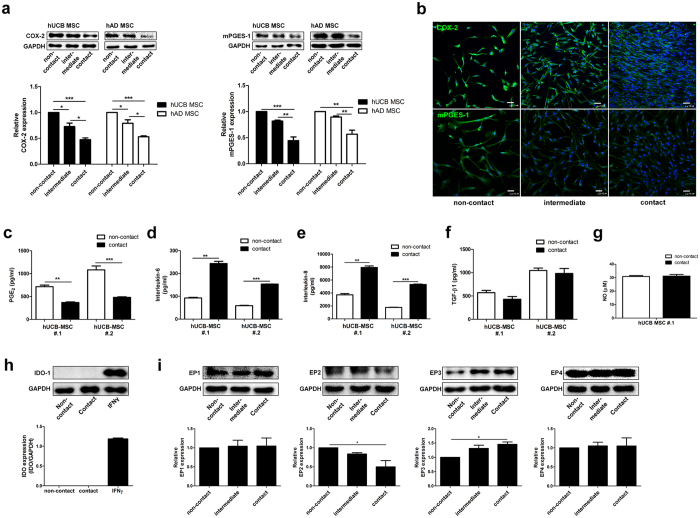
Cell contact regulates PGE_2_ secretion and expression of EP receptor in hMSCs. Identical numbers of cells were seeded on culture plates of different widths for 24 hours to achieve different cellular confluencies. (**a,b**) COX-2 and mPGES-1 protein levels were examined by (**a**) Western blot analysis and (**b**) immunocytochemistry. Bar = 500 μm. PGE_2_, IL-6, IL-8, TGF-β1, NO and IDO levels were determined from culture supernatant. (**c–g**) ELISA, (**h**) Western blotting of the IFNγ treatment group was used as a positive control. (**i**) Protein level of EP receptors was measured by Western blot analysis. Gel electrophoresis was conducted under the same experimental conditions, and images of blots were cropped. Uncropped blot images are shown in Suppl. Fig. S5. *P < 0.05, **P < 0.01, ***P < 0.001. Results are shown as the mean ± SEM.

Direct cell-to-cell contact between MSCs and immune cells is crucially involved in regulating the proliferation and activation of immune cells^15^,^27^. Although these cell contact-dependent regulatory mechanisms in MSC function have been reported by a number of groups, few mechanistic studies elucidate the effect of cell contact among the hMSCs themselves on their function. Therefore, we investigated whether cell contact among hMSCs can affect COX-2 and mPGES-1 protein expression as well as PGE_2_ secretion, which we proved to have a pivotal role in hMSC function. The COX-2 and mPGES-1 expression levels in both hUCB-MSCs and hAD-MSCs drastically decreased when the confluency of the same number of hMSCs was elevated by regulating the attachment area to allow cell-to-cell contact (Fig. 4a). These results were visually confirmed by the immunocytochemical staining of COX-2 and mPGES-1 in hUCB-MSCs plated at different confluencies. hUCB-MSCs plated at high density showed reduced expression levels of PGE_2_ synthesizing enzymes compared to cells with low density, the non-contact group (Fig. 4b). Subsequently, decreased COX-2 and mPGES-1 levels led to lower PGE_2_ secretion (Fig. 4c and Suppl. Fig. S4).

To determine whether the secretion profile of other soluble factors is affected by cell contact, levels of various cytokines were measured in the hUCB-MSC culture media. In contrast to PGE_2_, cell contact increased the production of interleukin (IL)-6 and IL-8, representative cytokines in NF-κB signalling (Fig. 4d,e). Secretion of prominent immunomodulatory factors from hMSCs, including transforming growth factor (TGF)-β1, nitric oxide (NO) and indoleamine-2,3-dioxygenase (IDO)-1, was not affected by cell contact (Fig. 4f–h and Suppl. Fig. S4).

Furthermore, the cell contact status altered the expression pattern of EP receptor subtypes. Western blot analysis showed that hMSCs expressed higher levels of EP2 receptor under non-contact conditions, whereas EP3 receptor expression increased with cell contact (Fig. 4i and Suppl. Fig. S4). These findings imply that cell-to-cell contact among hMSCs is important to regulate PGE_2_ secretion and EP receptor expression.

### Cell contact-dependent COX-2/PGE_2_ axis suppression is mediated by gap junction intercellular communication (GJIC)

**Figure 5:**
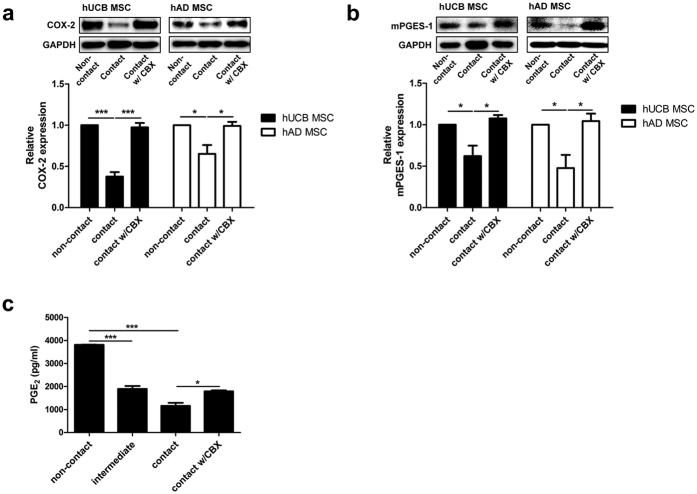
Gap junction intercellular communication (GJIC) is responsible for cellcontact-mediated PGE_2_ suppression in hMSCs. hMSCs under cell contact were treated with 100 μM carbenoxolone (CBX), a gap junction decoupler, for 24 hours. Protein expression levels of (**a**) COX-2 and (**b**) mPGES-1 were examined by Western blot analysis. (**c**) PGE_2_ concentrations were measured from the cultured media by ELISA. Gel electrophoresis was conducted under the same experimental conditions, and images of blots were cropped. Uncropped blot images are shown in Suppl. Fig. S5. *P < 0.05, ***P < 0.001. Results are shown as the mean ± SEM.

Gap junctions are formed when cells contact each other, and they regulate cellular function by allowing communication between adjacent cells. Therefore, we investigated whether gap junctions modulate the COX-2/PGE_2_ pathway by treating cells with the gap junction decoupler, carbenoxolone (CBX). hMSCs under contact conditions were treated with 100 μM carbenoxolone for 24 hours. Impaired expression of COX-2 and mPGES-1 in hMSCs with cell-to-cell contact was restored after CBX treatment to levels similar to those observed in the non-contact group (Fig. 5a,b). The restoration of the protein levels of these synthesis enzymes consequently led to increased PGE_2_ secretion (Fig. 5c). These findings indicate that PGE_2_ production is regulated by gap junction-mediated cell-to-cell interaction.

### PGE_2_ production is critical for the immunomodulatory ability of hMSCs, and cell contact-dependent inhibition of PGE_2_ release leads to the decline in this ability

**Figure 6:**
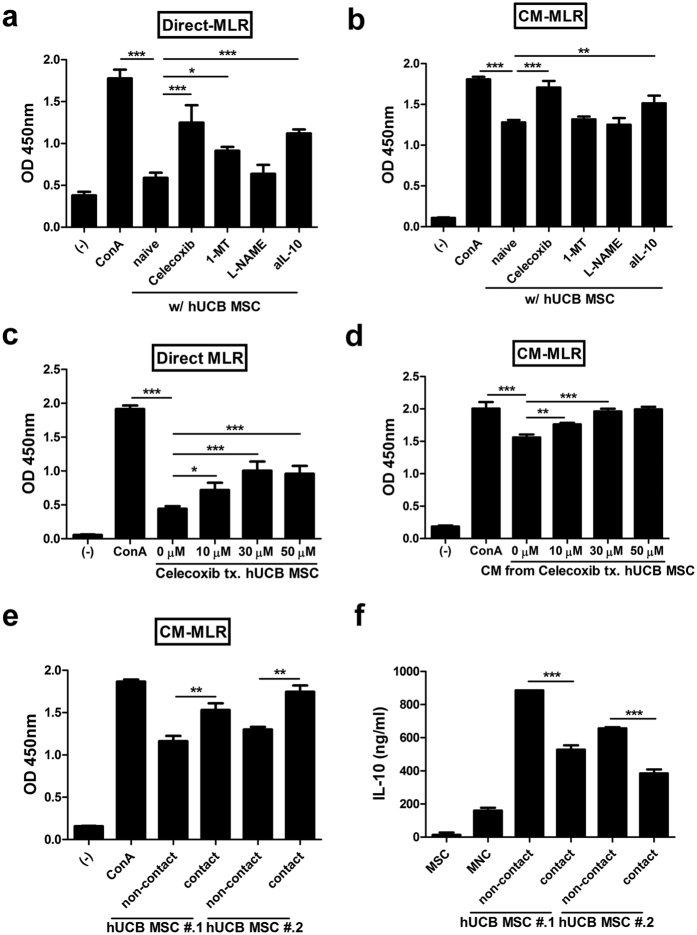
Cell contact-mediated decrease in PGE_2_ secretion is followed by the attenuation of the immunosuppressive effects of hMSCs. (**a–e**) Proliferation levels of mitogen-activated hMNCs were determined by BrdU, referred to as the MLR (mixed lymphocyte reaction) assay. (**a**) hMNCs were co-cultured with hUCB-MSCs that were pre-treated with selective inhibitors for various factors (Direct-MLR), or (**b**) cultured in the presence of conditioned media harvested from hUCB-MSCs (CM-MLR). Celecoxib: selective COX-2 inhibitor, 1-MT: selective IDO inhibitor, L-NAME: selective NOS inhibitor, aIL-10: neutralized with anti-IL-10 antibody. (**c**) hMNCs were co-culture with hUCB-MSCs treated with various doses of celecoxib, or (**d**) cultured in the presence of CM from hUCB-MSCs after treatment of various doses of celecoxib. (**e**) hMNCs were cultured in the presence of CM from the same number of hUCB-MSCs cultured under non-contact or contact condition. (**f**) IL-10 production in hMNCs cultured with CM from non-contact or contact hUCB-MSCs was measured by ELISA. *P < 0.05, **P < 0.01, ***P < 0.001. Results are shown as the mean ± SEM.

In our previous studies, we found that among soluble factors, PGE_2_ is a key molecule for the immunomodulatory function of hMSCs^15^,^17^. Therefore, we explored the significance of various soluble factors from hUCB-MSCs to inhibit mitogen-induced proliferation of mononuclear cells (MNCs). Mitogen-activated proliferation of MNCs was suppressed when they were co-cultured with hUCB-MSCs, and this inhibitory effect was reduced by the inhibition of COX-2, IDO-1 or IL-10 (Fig. 6a). When culture media (CM) from target factor-inhibited hUCB-MSCs was used to culture mitogen-treated MNCs, the inhibition of MNC proliferation in the CM was restored by suppressing COX-2 and IL-10 (Fig. 6b). Moreover, COX-2 inhibition in hUCB-MSCs by celecoxib treatment showed a dose-dependent decline in the suppression of MNC proliferation in both co-culture conditions allowing cell-to-cell contact and using CM (Fig. 6c,d).

Given that COX-2 signalling is pivotal in the immunomodulatory effect of hUCB-MSCs and that signalling can be altered by cell-to-cell contact, we further assessed whether cell contact status can modulate the immunosuppressive property of hUCB-MSCs. CM from the non-contact group inhibited the proliferation of MNCs to a greater extent than CM from the contact group (Fig. 6e). Moreover, the level of IL-10, a prominent anti-inflammatory cytokine, was elevated in the co-culture media, and hUCB-MSCs without cell contact exhibited more potent IL-10 production than cells with cell contact (Fig. 6f). Taken together, our findings suggest that PGE_2_ plays a crucial role in the immunosuppressive activity of hMSCs and that the cell contact regulation of PGE_2_ production in hMSCs correlates with their immune function.

## Discussion

Although recent studies have demonstrated that a number of autocrine signalling events are involved in the induction or maintenance of MSC functions such as proliferation, differentiation, migration and immunoregulation^28^,^29^,^30^,^31^,^32^, most of these studies focused on the elucidation of differentiation-related mechanisms. In the present study, we investigated the autocrine effect of PGE_2_ on MSCs proliferation, a major characteristic of stem cells that contributes to stemness. A few studies reported that hMSC proliferation is regulated by PGE_2_^25^,^26^. In these studies, hMSCs derived from bone marrow or umbilical cord blood were treated with various doses of PGE_2_. MSC proliferation consistently increased in response to PGE_2_ treatment via protein kinase A signalling in both studies. In the present study, we found that PGE_2_ produced by hUCB-MSCs and hAD-MSCs plays a crucial role in the maintenance of their proliferative function by regulating COX-2 signalling using chemical inhibitors or siRNA for COX-2. COX-2 inhibition by indomethacin or celecoxib treatment resulted in a consistent decrease in hMSC proliferation. This phenotype might result from the inhibition of potent signalling pathways in hMSCs via COX-2 signalling rather than PGE_2_, as COX-2/PGE_2_ signalling is involved in the several growth factor signalling pathways, including vascular endothelial growth factor and basic fibroblast growth factor^33^,^34^. We achieved similar effects by suppressing mPGES-1, an enzyme for PGE_2_ synthesis downstream of COX-2 signalling. Moreover, PGE_2_ treatment of COX-2-inhibited hMSCs rescued their proliferation in a dose-dependent manner. More importantly, we proved that secreted soluble factors from naïve hMSCs restored the proliferation of COX-2-inhibited hMSCs when they were co-cultured using a transwell system that prevented cell-to-cell contact, indicating that soluble factors from hMSCs themselves can contribute to their proliferation, presumably including PGE_2_. The previous study by Jang *et al.* showed that among the four major sub-types of E-type prostaglandin (EP) receptors, EP2 receptor has a pivotal role in PGE_2_-stimulated hUCB-MSC proliferation^25^. In this study, we demonstrated that only the selective antagonist for EP2 receptor down-regulated basal hMSC proliferation, implying that autocrine stimulation of PGE_2_ on hMSC proliferation is mediated by EP2 receptor. In addition, treatment with a selective agonist for EP2 receptor, butaprost, restored the proliferation of celecoxib-treated hMSCs.

Although a number of previous studies have shown that cell-to-cell contact between MSCs and immune cells or cancer cells is an important factor in the immunomodulatory or anti-tumour effect of MSCs, a few groups have focused on the cell-to-cell contacts between MSCs themselves and subsequent alterations in the secretion profile. Schajnovitz *et al.* reported that MSCs derived from bone marrow (BM-MSCs) possess functional gap junctions, and CXCL12 secretion by BM-MSCs is regulated by cell contact, leading to functional changes in MSCs to maintain the homeostasis of haematopoietic stem cells^35^. We show here that PGE_2_ secretion from the same number of MSCs was down-regulated when cell-to-cell contact was allowed, whereas production of IL-6 and IL-8 was increased, and TGF-β1 or NO production was not affected by confluent culture conditions that allow cell-to-cell contact. Decreased production of PGE_2_ exerted by cell contact was restored by the blockage of cellular communication using CBX, a well-known GJIC inhibitor, indicating that MSCs form functional syncytia via connexin gap junctions, leading to alterations in the secretion profile. In addition, previous studies from the Prockop group reported that compaction of hMSCs into spheroids self-activates signalling to enhance secretion of anti-inflammatory modulators such as PGE_2_, tumour necrosis factor α-induced protein 6 and stanniocalcin 1^36^,^37^. In these studies, hMSCs cultured as spheres using hanging drop culture produced markedly elevated levels of PGE_2_. This discrepancy in PGE_2_ regulation by cell contact might result from the differences in culture conditions for MSCs. In the present study, the general plastic adherent 2D culture method was used, and only the plating area was controlled to regulate cell-to-cell contact, whereas the hanging drop method to generate 3D spheroids was used in the studies by Prockop group.

PGE_2_ is a potent immunomodulator produced by hMSCs. hMSCs suppress the differentiation and maturation of T lymphocytes and induce the generation of regulatory T cells via COX2-mediated PGE_2_ secretion^27^,^38^,^39^. Moreover, PGE_2_ secretion from MSCs in response to certain inflammatory milieu critically contributes to the immunoregulatory function of MSCs against several immune disorders, including arthritis and colitis^15^,^40^. PGE_2_ produced by MSCs exerts anti-inflammatory effects through the regulation of immune cell activation and maturation, including CD4^+^ helper T cells, B cells, dendritic cells, natural killer cells, monocytes and macrophages^41^. In the present study, we have proven that cell contact-dependent regulation of PGE_2_ secretion correlates with the functional phenotype of MSCs. Importantly, down-regulation of PGE_2_ secretion by cell-to-cell contact led to a decreased immunomodulatory effect of MSCs in a mixed leukocyte reaction. Based on these findings, control of culture conditions regulating cell contact might be a crucial point in the development of therapeutics from MSCs, as a number of studies have been performed and are being conducted to produce therapeutics or cosmetics using conditioned media from MSCs^42^,^43^,^44^.

Taken together, the present study revealed novel information indicating that PGE_2_ secreted from adult stem cells exerts autocrine effects on MSC proliferation by triggering the EP2 receptor, and PGE_2_ production is dependent on cell-to-cell contact mediated by GJIC, resulting in a decline in immunoregulatory ability.

## Methods

### Isolation and culture of hUCB-MSCs

All experiments involving human umbilical cord blood (UCB) or UCB-derived cells were carried out in accordance with the approved guidelines of the Boramae Hospital Institutional Review Board (IRB) and the Seoul National University IRB (IRB No. 0603/001-002-10C4). The UCB samples were provided immediately after birth with informed consent and parent approval. The UCB from a donor was mixed with HetaSep solution (Stem Cell Technologies, Vancouver, Canada) at a ratio of 5:1 and incubated at room temperature for approximately one hour to remove red blood cells. Then, supernatant was collected using Ficoll, and mononuclear cells were separated after centrifugation at 2,500 rpm for 20 min. The cells were washed twice in phosphate-buffered saline (PBS). Isolated cells were seeded in growth media consisting of D-media (Formula No. 78–5470EF, Gibco BRL, NY, USA) containing EGM-2 SingleQuot and 10% foetal bovine serum (Gibco BRL, NY, USA). After 3 days, unattached cells were washed out, and adherent cell colonies were cultured to consistently establish sharp and spindle-shaped hUCB-MSCs. For the expansion of cells, KSB-3 complete media (Kangstem Biotech, Seoul, Korea) was used.

### Isolation and culture of hMNCs

The UCB samples were mixed with HetaSep solution (Stem Cell Technologies, Vancouver, Canada) at a ratio of 5:1 and incubated at room temperature for approximately one hour to remove red blood cells. Then, supernatant was collected with Ficoll, and mononuclear cells were separated after centrifugation at 2,500 rpm for 20 min. The cells were washed twice in PBS. Isolated cells were seeded in growth media consisting of RPMI 1640 (Gibco BRL, Grand Island, NY, USA) containing 10% foetal bovine serum.

### Isolation and culture of hAD-MSCs

All procedures using human adipose tissue or adipose tissue-derived mesenchymal stem cells were conducted in accordance with guidelines approved by Seoul National University IRB (IRB No. 0611/001-001). Freshly excised human mammary fat tissue, the waste from reduction mammoplasty, was digested for 2 hours with 1 mg/mL of type ΙA collagenase (≥125 CDU/mg solid, Sigma, St. Louis, MO, USA) at 37 °C. After washing in PBS and centrifugation at 1,000 rpm for 5 min, the tissue pellet was filtered through 100 μm nylon mesh and incubated in DMEM (Gibco BRL, NY, USA) containing 10% foetal bovine serum at 37 °C with 5% CO_2_. After 24 hours of incubation, unattached cells were washed out, and adherent cell colonies were cultured consistently in K-NAC media supplemented with 2 mM N-acetyl-L-cysteine (NAC; Sigma, St. Louis, MO, USA) and 0.2 mM L-ascorbic acid. For the expansion of cells, KSB-3 complete media (Kangstem Biotech, Seoul, Korea) was used.

### Reagents

Cay10526 and prostanoid receptor antagonists for EP1 (SC-51089) and EP2 (AH-6809) were purchased from Cayman Chemical Company (Ann Arbor, MI, USA). Celecoxib, butaprost, sulprostone, carbenoxolone, concanavalin A (ConA) from *Canavalia ensiformis* (Jack Bean) and the EP3 antagonist L-798106 were purchased from Sigma-Aldrich (St. Louis, MO, USA). The EP4 selective antagonist L-161,982 was purchased from Tocris Bioscience (Moorend Farm Ave., Bristol, UK). Recombinant human IFN-γ and TNF-α were obtained from Peprotech (Rocky Hill, NJ, USA).

### Western blot

The cells were washed twice in PBS and lysed in buffer containing 1% Nonidet-P40 supplemented with a complete protease inhibitor ‘cocktail’ (Roche, Indianapolis, IN, USA) and 2 mM dithiothreitol. The protein samples were separated by 10% SDS-PAGE and transferred to nitrocellulose membranes. After blocking with 3% bovine serum albumin (BSA) solution, proteins on the membrane were incubated with primary antibodies against COX-2, mPGES-1 (Abcam, Cambridge, MA, USA), EP1, EP2, EP3, EP4 (Cayman, Ann Arbor, MI, USA), iNOS and IDO-1 (Millipore, Billerica, MI, USA) more than 12 hours at 4 °C and then incubated with secondary antibodies. Protein and antibody complexes were detected using the ECL Western blotting detection reagent and analysis system.

### BrdU assay

For this assay, the cell proliferation ELISA kit (Roche, Indianapolis, IN, USA) was used. After the indicated treatment, cells were washed twice in PBS and incubated in growth media containing 100 μM bromodeoxyuridine (BrdU) labelling reagent for 2 hours at 37 °C in a humidified atmosphere with 5% CO_2_. After removing media and drying the cell surface, cells were fixed with the provided FixDenat solution for 30 minutes and incubated in peroxidase-conjugated anti-BrdU antibody (anti-BrdU-POD) solution for 90 minutes at room temperature. Cells were then washed three times in diluted washing solution and incubated with the provided substrate (tetramethyl-benzidine; TMB) solution for 5 to 30 minutes. After sufficient reaction and stop solution addition, the reaction products, which demonstrated cell proliferation levels, were quantified by measuring absorbance at the wavelength 450 nm and 690 nm (as a reference) using spectrophotometer.

### Cumulative population doubling level (CPDL) analysis

Cell proliferation was also measured by CPDL analysis. Estimated growth rates and proliferation levels were determined through the formula CPDL = ln (Nf/Ni) ln2, where Ni is the initial number of cells seeded, Nf is the final number of harvested cells, and ln is the natural log. First, 3 × 10^5^ cells isolated from different donors were seeded with or without indicated treatments, and the number of cells was counted after 3 to 5 days. Then, 3 × 10^5^ cells were seeded again with the same treatment. To determine the CPDL, population doublings for each passage were calculated and added.

### MTT assay

To indirectly assess cell viability and proliferation, an MTT assay was conducted. After treatment, cells were incubated in fresh medium containing 200 μg/ml of MTT reagent (Amresco, Solon, OH, USA) for 4 hours at 37 °C with 5% CO_2_. Medium was removed. DMSO was put into each well and incubated with shaking for approximately 2~3 min. The results were measured by an ELISA reader.

### Cell cycle assay

After indicated treatments, cells were harvested, washed twice in PBS, and fixed with ice-cold 70% ethanol at −20 °C for more than 30 min. Fixed cells were washed in PBS and incubated with 400 μl of PBS containing RNase A (7.5 μg/ml) and propidium iodide (50 μg/ml) at 37 °C for 30 min. Cell cycles were analysed by flow cytometry, which was performed on a FACScalibur using the Cell Quest software (BD Bioscience, San Jose, CA, USA).

### Apoptosis assay

For the apoptosis assay, commercially available Apoptosis Detection Kits (BD Bioscience, San Jose, CA, USA) were used. After indicated treatments, the cells were washed twice in PBS and resuspended in 100 μl of 1X binding buffer at a concentration of 1 × 10^5^. Then, 5 μl of FITC annexin V and 5 μl propidium iodide (PI) were added. The mixtures were gently vortexed and incubated for 15 min at room temperature in the dark. Then, 400 μl of 1X binding buffer was added to the mixtures, and all samples were analysed by flow cytometry, which was performed on a FACScalibur using Cell Quest software (BD Bioscience, San Jose, CA, USA).

### Immunocytochemistry

Cells at different confluencies were washed in PBS and fixed with 4% paraformaldehyde (PFA) at room temperature for 10 min. For permeabilization, the cells were incubated with 0.05% Triton X-100 solution at room temperature for 10 min and blocked with 5% normal goat serum (NGS) at room temperature for 1 hour. Then, the cells were stained with specific primary antibodies against COX-2 and mPGES-1 (Abcam, Cambridge, MA, USA) followed by 2 hours of incubation with Alexa 488-labelled secondary antibody (1:1,000; Molecular Probes, Eugene, OR, USA). The nuclei were stained with DAPI. The images were captured by a confocal microscope.

### Cytokine production

To determine the secretion level of various cytokines, culture supernatants were collected from cells incubated for 24 hours in non-contact or contact conditions. To determinate each concentration, commercial ELISA kits for PGE_2_, TGF-β1, IL-6, IL-8 (R&D Systems, Minneapolis, MN, USA) and NO (Cayman Chemical, Ann Arbor, MI, USA) were used according to the manufacturer’s protocols.

### Mixed leukocyte reaction

To collect culture supernatants (hUCB-MSC conditioned media; UCM), cells were incubated in non-contact or contact conditions for 24 hours and treated for 3 days with celecoxib in RPMI 1640 (Gibco BRL, Grand Island, NY, USA). Then, media were harvested after centrifugation. hMNCs prepared as described above were treated with ConA in collected culture supernatants for 5 days, and hMNC proliferation was determined by cell proliferation ELISA, BrdU kit (Roche, Indianapolis, IN, USA).

### Statistical analysis

Mean values of all results were expressed as the mean ± SEM. Statistical analyses were conducted using Student’s 2-tailed t-test or one-way ANOVA followed by Bonferroni post-hoc test for multigroup comparisons using GraphPad Prism version 5.0 (GraphPad Software, San Diego, CA, USA). Statistical significance is indicated in the figure legends.

## Additional Information

**How to cite this article**: Lee, B.-C. *et al.* PGE_2_ maintains self-renewal of human adult stem cells via EP2-mediated autocrine signaling and its production is regulated by cell-to-cell contact. *Sci. Rep.* **6**, 26298; doi: 10.1038/srep26298 (2016).

## Supplementary Material

Supplementary Information
