# Supplementary material for: PGE2 maintains self-renewal of human adult stem cells via EP2-mediated autocrine signaling and its production is regulated by cell-to-cell contact
Source: Sci Rep. 2016 May 27;6:26298. doi: 10.1038/srep26298 (PMC4882486; doi:10.1038/srep26298)
Supplement: Supplementary Information [file srep26298-s1.pdf]

Supplementary Information

PGE_2 maintains self-renewal of human adult stem cells via EP2-mediated autocrine signaling and its production is regulated by cell-to-cell contact

Byung-Chul Lee[1*], Hyung-Sik Kim[1,4,5*], Tae-Hoon Shin[1], Insung Kang[1], Jin Young Lee[1], Jae-Jun Kim[1], Hyun Kyoung Kang[1], Yoojin Seo[1], Seunghee Lee[2], Kyung-Rok Yu[1,3], Soon Won Choi[1,2], Kyung-Sun Kang[1,2†]

[1]Adult Stem Cell Research Center, College of Veterinary Medicine, Seoul National University, Seoul 08826, South Korea

[2]Research Institute for Veterinary Medicine, College of Veterinary Medicine, Seoul National University, Seoul 08826, South Korea

[3]Hematology Branch, National Heart, Lung and Blood Institute, National Institutes of Health, Bethesda, MD 20892, USA

[4]Pusan National University School of Medicine, Busan 49241, South Korea

[5]Biomedical Research Institute, Pusan National University Hospital, Busan 49241, South Korea

[*] These authors contributed equally to this work

[†]Correspondence

Kyung-Sun Kang, D.V.M., Ph.D.

Adult Stem Cell Research Center, College of Veterinary Medicine, Seoul National University,

1 Gwanak-ro, Gwanak-gu, Seoul 08826, South Korea

Tel. +82-2-880-1246

E-mail: kangpub@snu.ac.kr

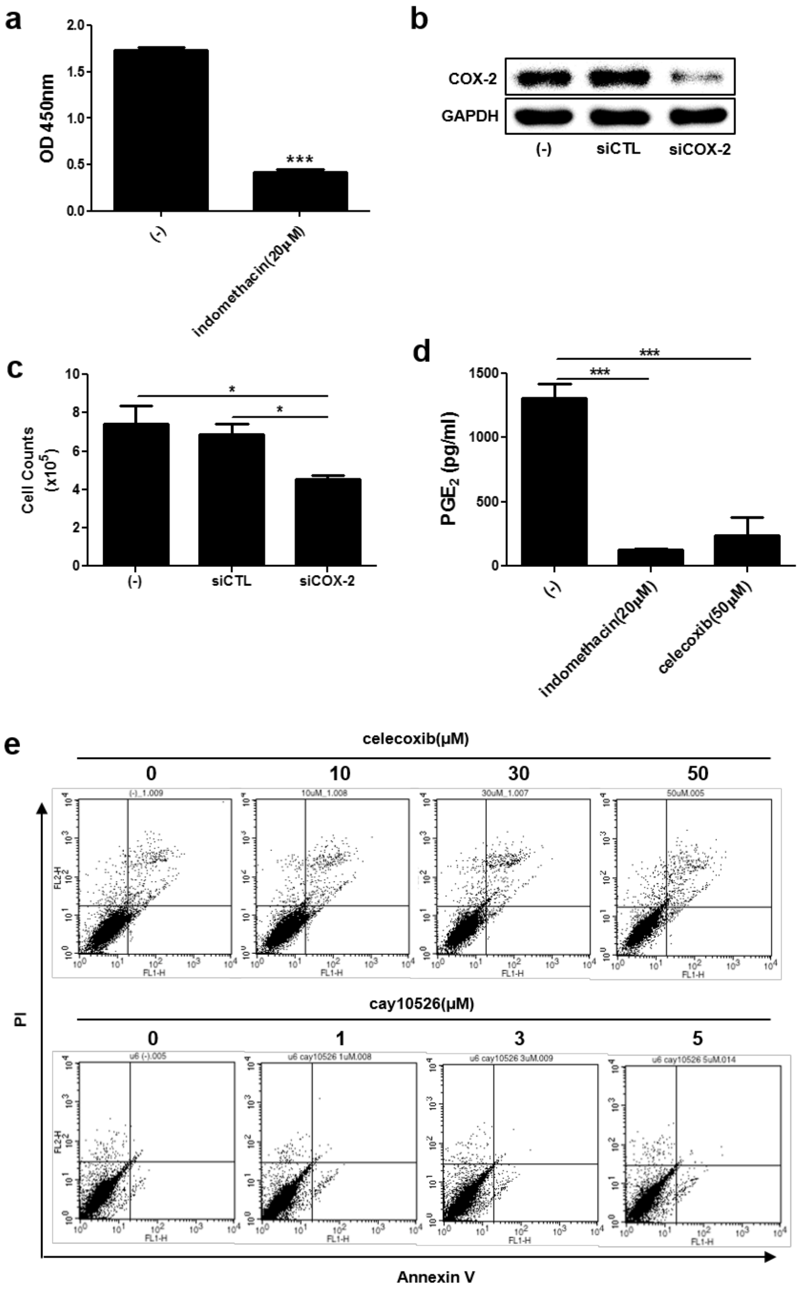

Supplementary Figure S1. MSCs proliferation is suppressed by the inhibition of COX-2 /PGE_2 axis (a) Proliferation of hUCB-MSCs treated with indomethacin, an inhibitor for both COX-1 and COX-2, was determined by BrdU kit. (b-c) hUCB-MSCs were transfected with siRNA for COX-2. (b) COX-2 expression on protein level, and (c) cell number were measured. (d) PGE_2 concentrations of indomethacin or celecoxib-treated cells were measured by ELISA. (e) Dot plot images of apoptosis assay. FL1; annexin V, FL2; PI (Propidium Iodide). Results show a representative experiment. * P<0.05, *** P< 0.001. Results are shown as mean ± SEM.

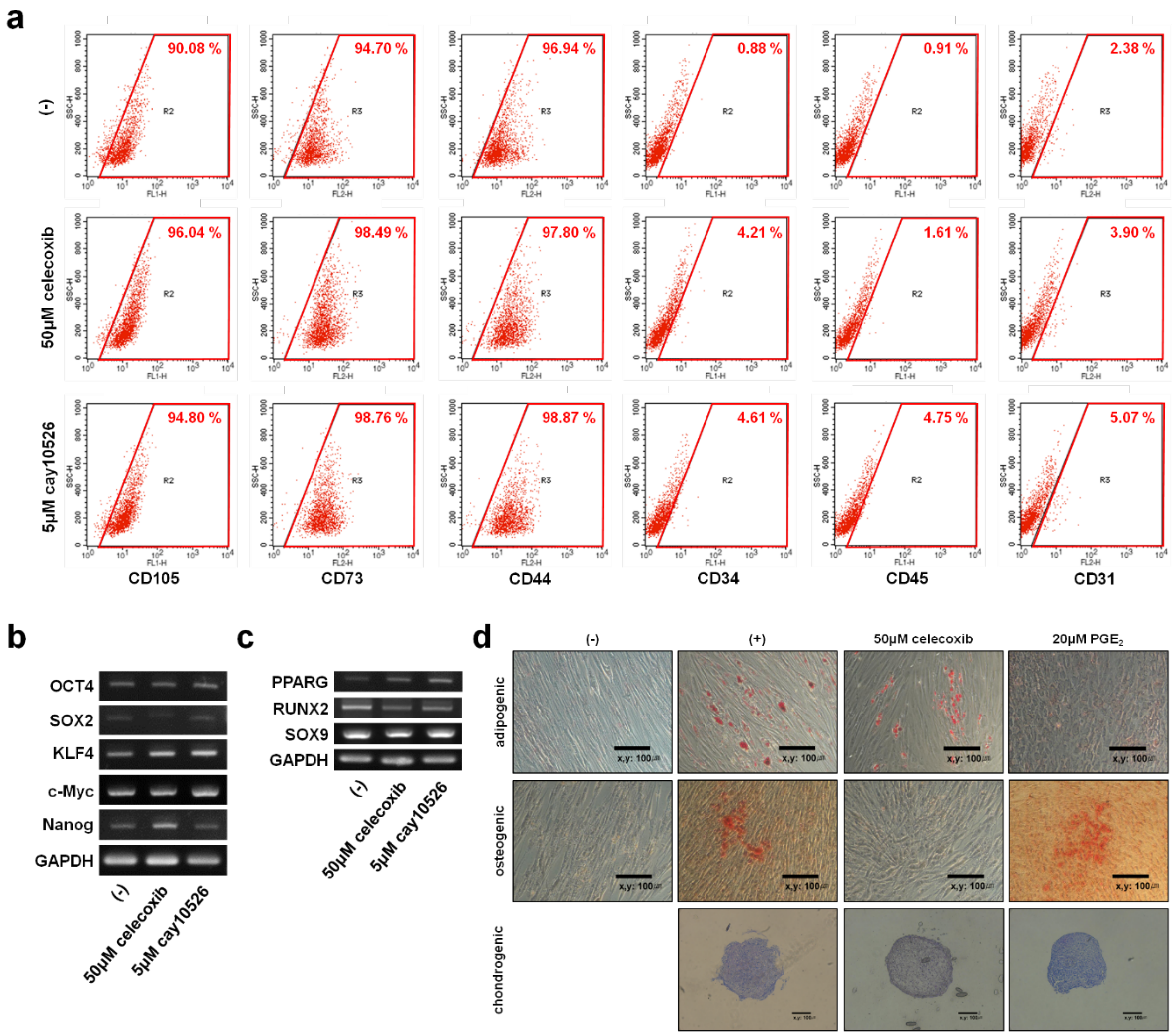

Supplementary Figure S2. Regulation of COX-2/PGE$_2$ axis alters the differentiation potential of hUCB-MSCs and does not influence the expression profiles of marker genes and CD markers. (a) The expression profiles of CD markers in hUCB-MSCs after the treatment of celecoxib or cay10526 were measured by flow cytometric analysis. Positive marker: CD105, CD73 and CD44, Negative marker: CD34, CD45 and CD31. (b-c) mRNA expressions of marker genes for (b) pluripotency and (c) differentiation in hUCB-MSCs were determined after treatment with celecoxib or cay10526 by RT-PCR. (d) hUCB-MSCs were differentiated into adipogenic-, osteogenic- and chondrogenic lineages in the presence of celecoxib and recombinant PGE$_2$. After 2 weeks of differentiation, the cells were stained with oil-red O for adipogenesis, alizarin S for osteogenesis and toluidine blue for chondrogenesis. Results show a representative experiment.

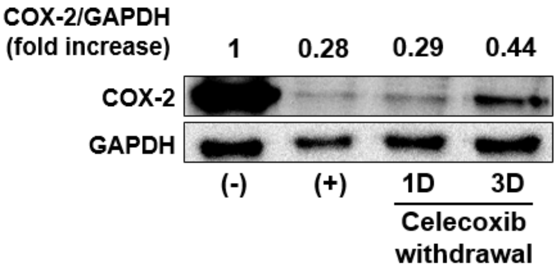

Supplementary Figure S3. Maintenance of COX-2 inhibition by celecoxib in protein level. At Day 1 and 3 after the celecoxib withdrawal, protein lysates are collected and COX-2 expression was measured on protein level by western blot analysis. Result shows a representative experiment.

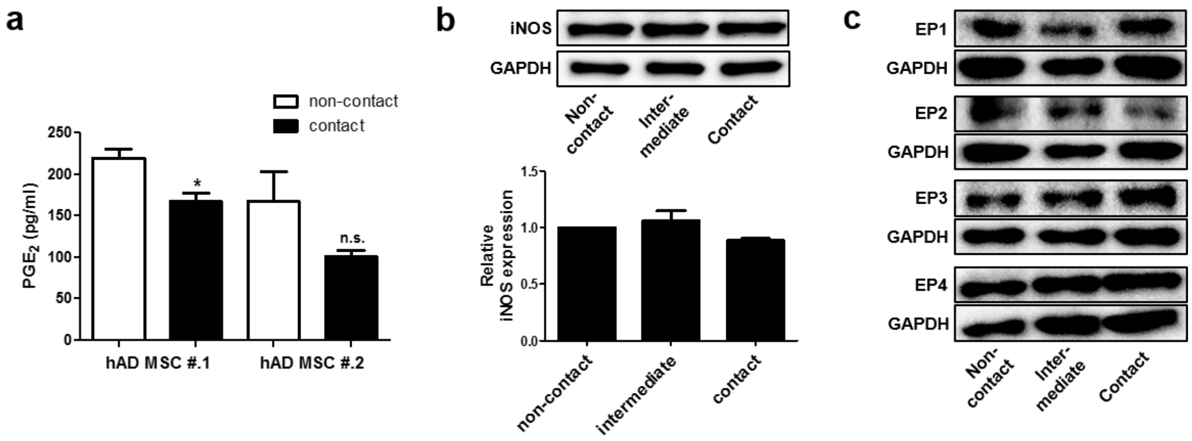

Supplementary Figure S4. Cell-to-cell contact regulates the expression of immunoregulatory soluble factors or their receptors in hMSCs. (a) PGE_2 secretion level of hAD-MSCs cultured in either non-contact or contact condition was determined by ELISA. (b) Cellular level of inducible NO synthase, iNOS of hUCB-MSCs was measured by western blot analysis. (c) Expression levels of EP receptors in hAD-MSCs were measured by western blot analysis. Results show a representative experiment. * $P<0.05$. Results are shown as mean ± SEM.

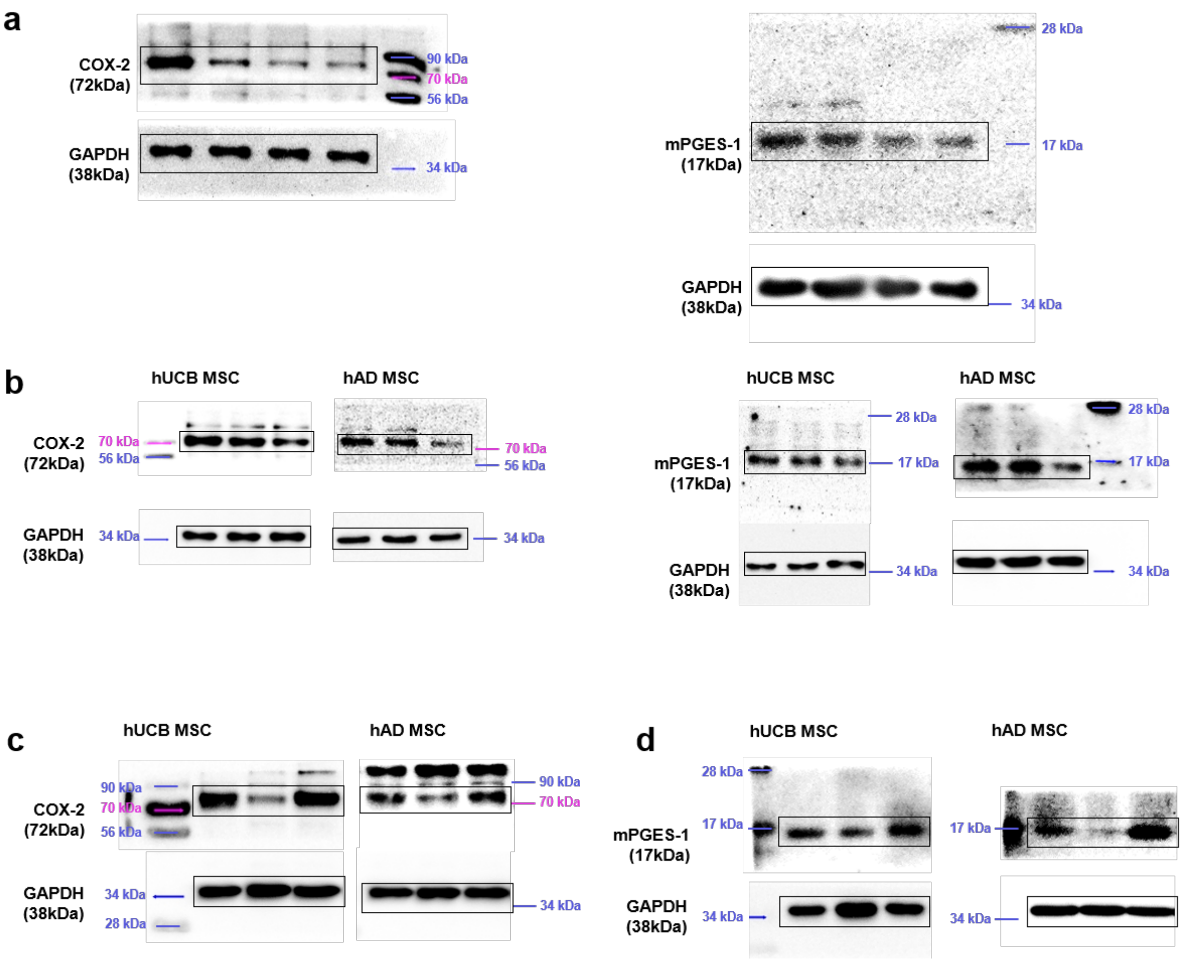

Supplementary Figure S5. Uncropped blot images of key data. Uncropped blot images of (a) Fig. 1a (b) Fig. 4a (c) Fig. 5a and (d) Fig. 5b. Gel electrophoresis was conducted under the same experimental conditions. Cropping lines are indicated.

Supplementary methods

Flow cytometric analysis

hUCB-MSCs (1x10^6 cells) were stained with FITC- or PE-conjugated antibodies specific for human CD31, CD34, CD44, CD45, CD72 and CD105. Non-specific isotype-matched antibodies used for negative controls. All the antibodies were purchased from BD Bioscience (San Jose, CA, USA), the analysis was performed on a FACS calibur using Cell Quest software (BD Bioscience, San Jose, CA, USA).

RNA extraction and RT-PCR

Total RNA was extracted from hUCB MSCs using 1 ml of Trizol reagent (Invitrogen, Carlsbad, CA, USA) according to the manufacturer’s protocol. cDNA was prepared from 1 μg of total RNA by using the Superscript III First-Strand Synthesis System (Invitrogen, Carlsbad, CA, USA). For RT-PCR, the cDNA and primers were combined with a PCR premix (Bioneer, Seongnam, Korea) and the PCR products were separated on a 1.5% agarose gel, visualized, and photographed using a gel documentation system.

Differentiation assay

For adipogenic and osteogenic differentiation, hUCB-MSCs were plated in six-well plate at 70-80% confluence and after stabilization more than 12 hours, cultured with adipogenic differentiation medium (DMEM supplemented with 10% FBS, 1 μM dexamethasone, 10 μM insulin, 200 μM indomethacin and 0.5 mM isobutylmethylxanthine; IBMX) or an osteogenic differentiation medium (DMEM supplemented 10% FBS, 50 μM ascorbic acid, 0.1 μM dexamethasone, 10 mM β-glycerophsphate). DMEM supplemented with 10% FBS was used as a control and media were replaced twice a week. After 2 weeks of induction, adipogenesis

of the cells were determined by staining intracellular lipid accumulation with Oil Red O, osteogenesis were visualized with Alizarin Red staining which is specific for calcium.

For chondrogenic differentiation, 5x105 cells were seeded in 15 ml polypropylene tube and centrifuged to form cell pellets. The pellets were incubated with 1 ml of chondrogenic differentiation medium (Lonza, Allendale, NJ, USA) and the medium replaced twice a week. After 3 weeks, the pellets were fixed, processed, embedded, and sliced into 3 μm sections. The sections were stained with toluidine blue following general procedures.
